# Expression of Osteopontin and Gremlin 1 Proteins in Cardiomyocytes in Ischemic Heart Failure

**DOI:** 10.3390/ijms25158240

**Published:** 2024-07-28

**Authors:** Milda Kuprytė, Vaiva Lesauskaitė, Vitalija Siratavičiūtė, Lina Utkienė, Lina Jusienė, Dalia Pangonytė

**Affiliations:** 1 Laboratory of Cardiac Pathology, Institute of Cardiology, Lithuanian University of Health Sciences, LT-50161 Kaunas, Lithuania; milda.kupryte@lsmuni.lt (M.K.); vitalija.sirataviciute@lsmuni.lt (V.S.); lina.utkiene@lsmuni.lt (L.U.); lina.jusiene@lsmuni.lt (L.J.); 2 Laboratory of Molecular Cardiology, Institute of Cardiology, Lithuanian University of Health Sciences, LT-50161 Kaunas, Lithuania; vaiva.lesauskaite@lsmuni.lt

**Keywords:** osteopontin, gremlin 1, immunohistochemistry, cardiomyocyte remodeling, ischemic heart failure

## Abstract

A relevant role of osteopontin (OPN) and gremlin 1 (Grem1) in regulating cardiac tissue remodeling and formation of heart failure (HF) are documented, with the changes of OPN and Grem1 levels in blood plasma due to acute ischemia, ischemic heart disease-induced advanced HF or dilatative cardiomyopathy being the primary focus in most of these studies. However, knowledge on the early OPN and Grem1 proteins expression changes within cardiomyocytes during remodeling due to chronic ischemia remains insufficient. The aim of this study was to determine the OPN and Grem1 proteins expression changes in human cardiomyocytes at different stages of ischemic HF. A semi-quantitative immunohistochemical analysis was performed in 105 myocardial tissue samples obtained from the left cardiac ventricles. Increased OPN immunostaining intensity was already detected in the stage A HF group, compared to the control group (*p* < 0.001), and continued to increase in the stage B HF (*p* < 0.001), achieving the peak of immunostaining in the stages C/D HF group (*p* < 0.001). Similar data of Grem1 immunostaining intensity changes in cardiomyocytes were documented. Significantly positive correlations were detected between OPN, Grem1 expression in cardiomyocytes and their diameter as well as the length, in addition to positive correlation between OPN and Grem1 expression changes within cardiomyocytes. These novel findings suggest that OPN and Grem1 contribute significantly to reorganization of cellular geometry from the earliest stage of cardiomyocyte remodeling, providing new insights into the ischemic HF pathogenesis.

## 1. Introduction

Heart failure (HF) is a clinical syndrome associated with inadequate cardiac output due to structural and functional abnormalities, with an ischemic origin being one of the principal causes for HF [1]. As a result of improved diagnostic and treatment strategies, the age-adjusted incidence of HF is declining [2], but the total number of patients living with this diagnosed cardiac pathology is still increasing with an estimated 64 million HF patients worldwide, causing a significant medical and economic burden [1]. Although general tendencies of HF pathogenesis mechanisms are well established, studies show that these mechanisms do not adequately address the highly complicated nature of remodeling processes occurring due to ischemic injuries in cardiac muscle until ischemic HF develops. While inducing intracellular protein expression changes in cardiomyocytes of both ischemic and non-ischemic regions of cardiac ventricles, remodeling provides the cellular plasticity to adapt, survive, and regain functionality of the damaged tissue, resulting in changes of the cardiac chamber size, shape, and function [3]. Nonetheless, when these intracellular adaptive mechanisms are exhausted, cardiomyocyte’s ability to maintain functionality starts to become disrupted, leading to the deterioration of the whole cardiac muscle tissue, and eventually progressing towards symptomatic HF [4].

Considering the established cardiomyocyte’s role in remodeling and the increasing focus on the changes in intracellular protein expression during the remodeling in myocardial tissue, a scientific interest has emerged focusing on the easily identifiable and representative protein markers to observe the actual reorganization of protein expression changes in cardiomyocytes [4,5,6]. Osteopontin (OPN), a matricellular phosphorylated glycoprotein [7,8] and a proposed therapeutic HF target [9], is known to balance inflammatory processes, induce expression of genes responsible for cardiomyocyte hypertrophy [10,11,12], and participate in intercellular communication [13]. Moreover, significant positive correlative trends between the OPN levels in plasma and the deteriorating cardio-metabolic function, severity of cardiac ventricular dysfunction are described [6,14,15]. Also, gremlin 1 (Grem1), a bone morphogenetic protein antagonist of the Dan protein family, crucial in the embryogenesis of the heart [16,17], regulation of specific immune response reactions, remodeling of the tissues [18], and modulation of angiogenesis in hypoxic microenvironment [19], demonstrates diagnostic potential in clinical follow-up and outcome prognosis for acute HF [13].

Studies demonstrate that the OPN and Grem1 expression level changes observed during remodeling can contribute towards determining the cellular functionality potential, while also revealing the new aspects of HF pathogenesis mechanisms and serving as validated diagnostic tools for the detection of therapeutic targets to sustain functionality of cardiac myocytes [9,20]. Also, accurate and quantifiable data on the OPN and Grem1 expression in cardiomyocytes can contribute to the stratification of different risk group patients, follow-up of the HF disease progress, and the evaluation of individualized HF treatment strategy [21,22,23,24].

However, despite extensively characterized clinical significance of OPN and Grem1 changes in ischemic HF-associated cardiac injury context, most of the studies present correlative analysis of OPN, Grem1 expression changes and clinical parameters of progressing cardiac dysfunction relying on the blood plasma levels of these proteins [6,13,21,22], without documenting the actual primary source of these changes within myocardial tissue. Notably, a small number of studies focusing on immunohistochemical OPN and Grem1 expression in myocardial tissue cells exist. Still, most of these studies principally describe OPN and Grem1 expression changes in cardiomyocytes that are exposed to dilatative cardiomyopathy, acute ischemia or ischemic heart disease-induced advanced HF [11,23,25,26]. Characterization of the OPN and Grem1 proteins expression changes in cardiomyocytes at the early stages of ischemic HF, crucial for extending the knowledge on the OPN and Grem1 roles in the earliest events within failing cardiomyocytes in ischemic HF pathogenesis and searching for the novel diagnostic or therapeutic targets, remains principally out of scope in these studies. Moreover, most of the studies on OPN and Grem1 expression in HF are largely limited on a single, mostly symptomatic, end-stage HF in animal or cell culture models without control groups as the major limitations of diagnostically applicable results [23,24,25,27,28]. Therefore, OPN and Grem1 proteins expression data on the earliest events within failing cardiomyocytes during remodeling in myocardial tissue exposed to continuous ischemia before symptomatic ischemic HF in the human organism remain obscure.

This study aimed to detect and document the significant expression changes of OPN and Grem1 proteins in cardiomyocytes at different stages of ischemic HF, including stage A (at-risk for HF), stage B (pre-HF), and stages C/D HF (symptomatic and advanced HF) according to the American College of Cardiology (ACC)/American Heart Association (AHA) classification [29], by applying validated semi-quantitative immunohistochemical evaluation. Considering the OPN and Grem1 proteins roles in HF pathogenesis, it was hypothesized that significant changes of OPN and Grem1 proteins expression in cardiomyocytes can be already observed at the earliest stages of ischemic HF before the first clinical HF symptoms appear. Further, we have explored the correlation between expression changes of OPN as well as Grem1 proteins in cardiomyocytes and their cellular geometric parameters, further illustrating the roles of OPN and Grem1 proteins in the earliest events of cardiomyocyte remodeling contributing to advancing ischemic HF. Importantly, experimental animal model data on OPN and Grem1 expression in failing myocardium are translated into the clinical setting by testing the study hypothesis of OPN and Grem1 proteins expression in cardiomyocytes of human myocardial tissues.

## 2. Results

### 2.1. OPN Expression

**Figure 1:**
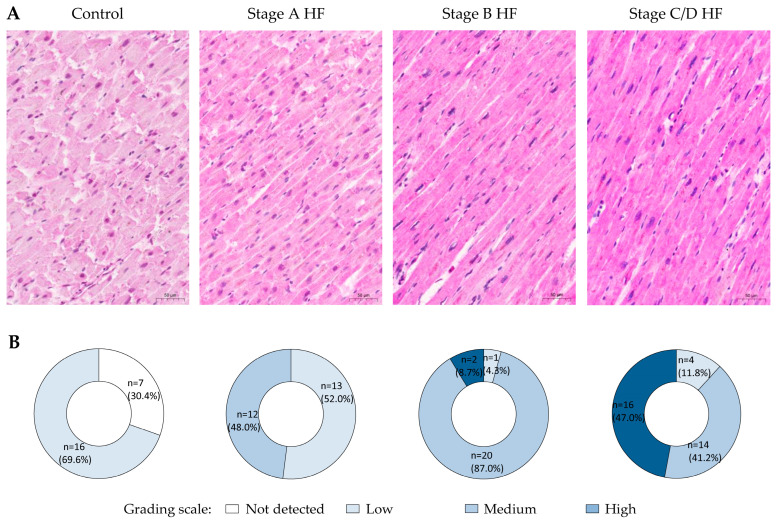
Expression patterns of OPN in cardiomyocytes of different groups. (**A**) Representative images of myocardium immunohistochemistry (OPN, MPIIIB10(1)). (**B**) Pie charts depict group stratification according to the grading scale of immunohistochemical reaction in cardiomyocytes. Abbreviations: HF—heart failure; stages A, B, C, D of HF—according to ACC/AHA classification. Scale bar: 50 µm.

A semi-quantitative analysis of immunohistochemical reaction against OPN revealed that the low level of OPN staining was predominating in the control group, with approximately one-third of cases documented with no detected OPN immunostaining in cardiomyocytes at all (Figure 1).

When analyzing OPN immunostaining in cardiomyocytes of the stage A HF group, almost half of the cases within this group demonstrated medium level of OPN expression, while low level of OPN staining was observed in cardiomyocytes of remaining cases. A small number of cases with the high OPN grading scale category was first observed in the stage B HF group, where the medium level of OPN immunostaining in cardiomyocytes was predominant in more than 85% of the cases within this group. The high level of OPN immunostaining in cardiomyocytes was also detected in the stages C/D HF group, where this grading scale category was observed in almost half of the cases within this group, while more than one-third of cases demonstrated medium level of OPN immunostaining in the same group.

**Figure 2:**
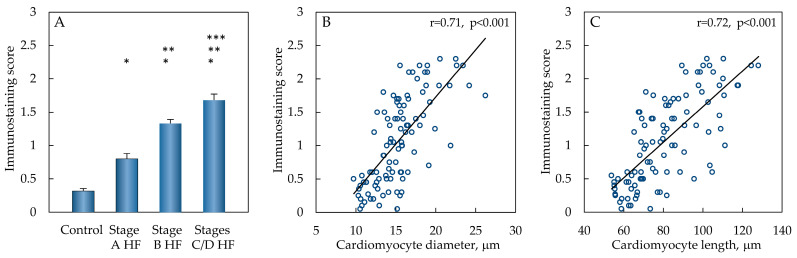
OPN expression in different groups. Semi-quantitative analysis results of OPN immunostaining (**A**), data are presented as mean and standard error: * *p* < 0.001—stage A HF, stage B HF, and stage C/D HF groups compared to the control group; ** *p* < 0.001—stage B HF and stage C/D groups compared to stage A HF group; *** *p* < 0.001—stages C/D HF group compared to stage B HF group (ANOVA with post hoc Bonferroni tests for multiple comparisons). Correlations of OPN immunostaining score and cardiomyocyte diameter (**B**), OPN immunostaining score and cardiomyocyte length (**C**). Abbreviations: HF—heart failure; stages A, B, C, D of HF—according to ACC/AHA classification; µm—micrometer; r—Pearson’s correlation coefficient.

When comparing the OPN immunostaining score, this score increased significantly in the stage A HF group compared to the control group (*p* < 0.001) (Figure 2). OPN immunostaining score continued to increase in the stage B HF group and was significantly higher compared to the control (*p* < 0.001) and stage A HF groups (*p* < 0.001). The highest immunostaining score of OPN expression in cardiomyocytes was detected in the stages C/D HF group, which was significantly increased compared to the control (*p* < 0.001), stage A HF (*p* < 0.001), and stage B HF groups (*p* < 0.001).

While exploring the associative trends of OPN expression in cardiomyocyte and the cardiac myocyte’s geometric parameters, strong positive correlations were detected between the OPN expression in cardiomyocyte’s cytoplasm and its cellular diameter (r = 0.71, *p* < 0.001) as well as in the intracytoplasmic OPN expression in cardiac myocytes and the cardiomyocyte length (r = 0.72, *p* < 0.001) (see Figure 2).

### 2.2. Grem1 Expression

**Figure 3:**
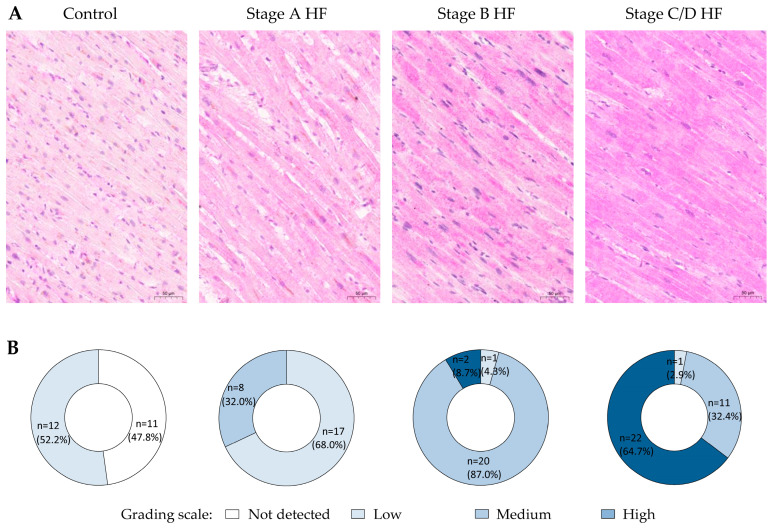
Expression patterns of Grem1 in cardiomyocytes of different groups. (**A**) Representative images of myocardium immunohistochemistry (Grem1, ab22138). (**B**) Pie charts depict group stratification according to the grading scale of immunohistochemical reaction in cardiomyocytes. Abbreviations: HF—heart failure; stages A, B, C, D of HF—according to ACC/AHA classification. Scale bar: 50 µm.

A low level of Grem1 immunostaining was predominating in more than half of the cases within the control group (Figure 3). Notably, no detectable Grem1 immunostaining in cardiomyocytes was observed in almost half of the control group cases. The medium level of grading scale category was detected in more than one-fourth of the stage A HF group cases, whereas the remaining cases demonstrated predominantly weak immunostaining intensity of Grem1 in cardiomyocytes. Interestingly, the high level of Grem1 was first observed in almost one-tenth of the cases of the stage B HF group, where the medium level of Grem1 immunostaining grading scale was documented in more than 80% cases within this group. The most abundant number of cases presenting a high level of Grem1 immunostaining intensity was observed in the stages C/D HF group with more than two-thirds of the analyzed cases demonstrating predominantly strong Grem1 immunostaining pattern.

**Figure 4:**
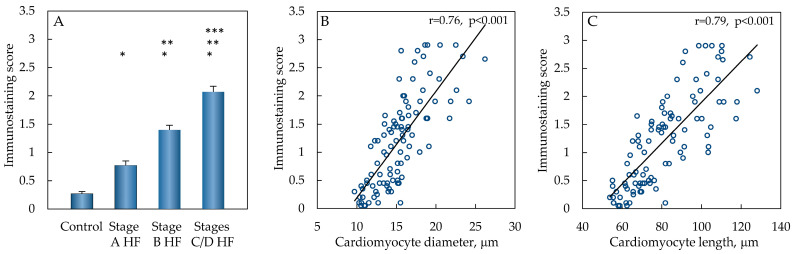
Grem1 expression in different groups. Semi-quantitative analysis results of Grem1 immunostaining (**A**), data are presented as mean and standard error: * *p* < 0.001—stage A HF, stage B HF, and stage C/D HF groups compared to the control group; ** *p* < 0.001—stage B HF and stage C/D groups compared to stage A HF group; *** *p* < 0.001—stages C/D HF group compared to stage B HF group (ANOVA with post hoc Bonferroni tests for multiple comparisons). Correlations of Grem1 immunostaining score and cardiomyocyte diameter (**B**), Grem1 immunostaining score and cardiomyocyte length (**C**). Abbreviations: HF—heart failure; stages A, B, C, D of HF—according to ACC/AHA classification; µm—micrometer; r—Pearson’s correlation coefficient.

Furthermore, a significantly increased Grem1 immunostaining score was detected in the stage A HF group compared to the control group (*p* < 0.001) (Figure 4). Even higher value of Grem1 immunostaining score was documented in the stage B HF group, when comparing this parameter to the control (*p* < 0.001) and stage A HF groups (*p* < 0.001). Immunostaining score of Grem1 expression in cardiomyocytes continued to increase in the stages C/D HF group, where the highest value of this score was observed, comparing this Grem1 immunostaining score to the control (*p* < 0.001), stage A HF (*p* < 0.001), and stage B HF groups (*p* < 0.001).

Analysis of the correlations between intracytoplasmic Grem1 expression in cardiac myocytes and their diameter as well as the length was performed, and a strong positive correlation was detected between the Grem1 immunostaining score and the cardiomyocyte diameter (r = 0.76, *p* < 0.001), as well as between the Grem1 immunostaining score and the cellular length of cardiac myocyte (r = 0.79, *p* < 0.001) (see Figure 4).

**Figure 5:**
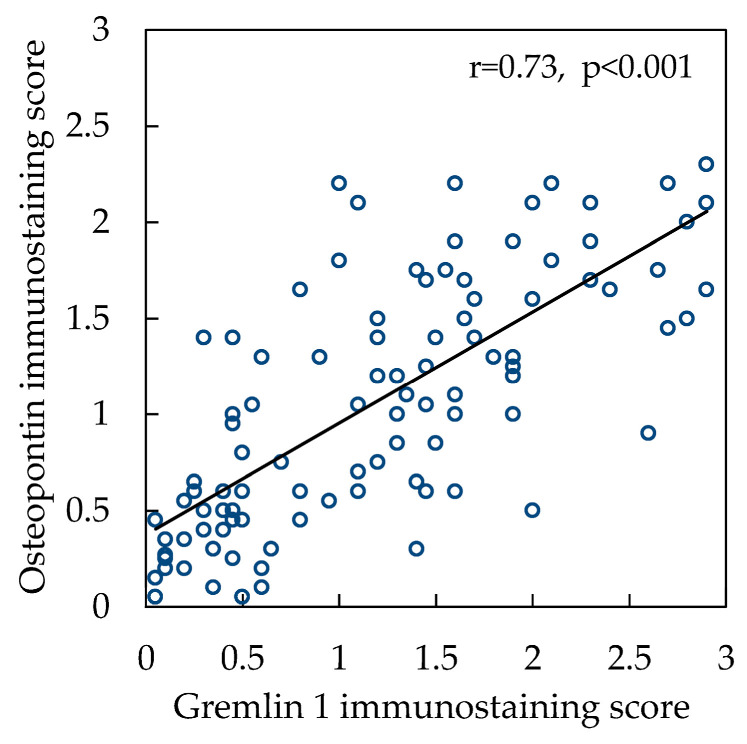
Correlation between OPN and Grem1 immunostaining scores. Abbreviation: r—Pearson’s correlation coefficient.

Interestingly, analysis of the correlation between OPN and Grem1 immunostaining scores demonstrated a significantly strong positive correlation of these proteins’ expression in the cardiac myocytes (r = 0.73, *p* < 0.001) (Figure 5).

## 3. Discussion

OPN is a protein with widely analyzed and acknowledged sensitive diagnostic properties [30], when it comes to different pathologies of irreversibly deteriorating cardiac muscle with progressing HF due to dilatative cardiomyopathy [31], diabetic cardiomyopathy [6], or ischemic heart disease [22,32]. Significant correlative tendencies of OPN and echocardiography parameters, HF course, outcomes, hospitalization, prognostic as well as predictive indicators are documented [30,32]. Behnes M. et al. noticed that plasma levels of OPN were equivalent with echocardiographic left cardiac ventricular parameters and correlated positively with the functional New York Heart Association (NYHA) classification as well as with the structural classification of HF by the ACC/AHA classification in acute HF [30], furthermore emphasizing the overall biological importance of OPN in HF pathogenesis. Still, most of these studies focus on OPN changes measuring the OPN concentration levels in blood plasma, while setting the pathological values of this parameter based on the clinical correlations with HF which is already advanced [22].

To the best of our knowledge, our study is the first attempt to present an extensive characterization of OPN protein expression dynamics in cardiomyocytes, validated by immunohistochemical staining in the different stages of ischemic heart disease-induced HF. Our study is the first one to describe a significantly increased OPN protein expression in cardiomyocytes in the stage A ischemic HF (at-risk for HF), OPN immunostaining intensity evaluated in cardiomyocytes continued to increase significantly at the stage B HF (pre-HF). The predominantly highest level of OPN immunoreactivity was documented in cardiac myocytes at the stages C/D HF (symptomatic and advanced HF). A significantly increased OPN expression was detected in ischemic heart disease-induced HF within sudden cardiac death cases of diabetes mellitus patients compared to the control group in the study by Patel M. et al. [8]. Also, Schipper M. E. I. et al. documented that expression of OPN mRNA as well as the OPN protein in cardiomyocytes of ischemic heart disease-induced advanced HF was significantly decreased after the implantation of left ventricular assist device [23]. These findings of OPN expression changes in cardiac myocytes correlating with progressing cardiac muscle dysfunction suggest that OPN may be directly involved in disrupting the contractile function of the cardiac muscle exposed to ischemia, leading to maladaptive changes of cardiac ventricular geometry, and, eventually, to advanced ischemic heart disease-induced HF.

A correlative trend of OPN expression and the geometric parameters of cardiomyocyte, representing cardiac ventricular geometry changes on a macroscopic scale, revealed significant positive correlative trends between OPN expression in the cardiomyocytes and cellular diameter as well as cellular length of the cardiac myocytes when myocardium is exposed to ischemic injury. To our knowledge, this is the first time such correlations between the OPN protein expression changes within cardiomyocytes and cardiac myocyte geometry are characterized in myocardial tissue exposed to continuous ischemia. Associative trends between the enhanced OPN expression and cardiomyocyte hypertrophy both in vivo and in vitro were already characterized by Graf et al. in systemic arterial hypertension on animal experiments [11]. The study of OPN expression in dilatative cardiomyopathy demonstrated that the increasing OPN expression in cardiac myocytes was observed simultaneously with the increasing mean diameter of cardiomyocyte in advanced HF (r = 0.731, *p* < 0.001) [25], corresponding to the similar data in our study. Furthermore, Li J. et al. determined that significant dynamic changes of OPN expression and cellular morphometric changes start manifesting quite early after exposure to the injuring factor, with less significant dynamic changes of OPN expression being apparent after 12 weeks of implementing a pressure overload-induced HF experimental model on animals [9], supporting the fact of OPN biological significance at the early stages of cardiomyocyte injury and its effect on cellular geometry changes.

OPN, as a matricellular protein, modulates the processes of intercellular communication, cellular adhesion, and migration under physiological circumstances, and its expression in non-injured cardiomyocytes is documented as low [10], corresponding to the findings of OPN protein expression in the control group of our study. However, when the cardiomyocyte is exposed to ischemic conditions, a more extensive scope of OPN functions is promoted immediately. Initiated intracellular signaling pathways, such as CD44/RAF/RAS/MEK1-2/ERK1-2 or calcineurin/NFAT, lead to the activation of the cellular hypertrophy-inducing genes expression [33,34], eventually manifesting as a compensatory hypertrophy in the cardiac myocyte to serve as a protective mechanism against ischemic cellular injury. Since non-detectable OPN expression in cardiomyocytes was observed exclusively in the control group of our study, it can be hypothesized that the significantly increased OPN expression in cardiomyocytes already observed at the stage A HF of our study demonstrates how sensitive and balanced cellular molecular mechanisms of the remodeling are from the very beginning to react and compensate immediately for any intracellular dysfunctions caused by ischemia.

As remodeling of cardiomyocytes induced by chronic ischemia continues, OPN expression in cardiomyocytes continues to increase simultaneously in stage B ischemic HF until it reaches a peak of this protein expression in symptomatic and advanced stages of ischemic HF observed in our study. These findings indicate the more complex biological role of OPN in cardiac myocyte remodeling when cardioprotective aspect of OPN functions is lost in advancing ischemic injury of failing cardiac muscle. This can be explained by results of Dalal S. et al. study where researchers revealed that OPN overexpression in adult mice cardiac myocytes leads to increased cardiomyocyte apoptosis via CD44-mediated mitochondrial death pathways and endoplasmic reticulum stress [35]. Also, decrease of cardioprotective OPN effect can be explained by direct inhibitory role of OPN in cardiac β2AR anti-fibrotic signaling via cAMP/Epac1 pathway described by Pollard C.M. et al. [36]. Together, these mechanisms modulated by changes in cardiomyocyte OPN expression affect functional cardiac ventricular geometry by increasing cardiac wall mass, and ventricular enlargement. Eventually these processes contribute to the increased systolic longitudinal wall stress and global ventricular dysfunction, clinically advancing towards symptomatic HF [37]. Psarras S. et al. determined an increase of the posterior left cardiac ventricular wall thickness by 30% together with an increase of the systolic left cardiac ventricular function by 53% and a decrease of the left cardiac ventricular dilation by 29% with almost normalized end-diastolic diameter values in OPN^-/-^ mutated mice with dilatative cardiomyopathy [38], further proving OPN’s role in adverse cardiomyocyte remodeling and pathological changes of cardiac ventricular geometry. As a result of decreasing cardiac output, renin–angiotensin–aldosterone system is also activated, further affecting the size of cardiomyocytes, and increasing activity of fibroblasts, thus promoting myocardial stiffness and diastolic dysfunction while progressing to the advanced HF [39].

While representing a more detailed characterization of the processes in cardiomyocytes advancing towards ischemic HF, a significant increase of Grem1 expression in cardiomyocytes was already detected in the ischemic at-risk for HF group compared to the control group in our study. Similarly to OPN expression in cardiomyocytes, Grem1 expression was found to be even more increased in cardiomyocytes of pre-HF group. The highest level of Grem1 immunostaining was detected in the symptomatic and advanced HF group, determining significant differences of Grem1 expression when comparing the results of Grem1 immunostaining analysis to the control, at-risk for HF, and pre-HF groups. Since data on Grem1 expression in cardiomyocytes exposed to ischemia is scarce, these findings firstly presented in our study represent a unique morphological insight of Grem1 expression changes in cardiomyocytes of deteriorating cardiac muscle, advancing towards symptomatic ischemic heart disease-induced HF.

Characterized findings of Grem1 protein expression in cardiomyocytes documented in our study were consistent with study results of Mueller A. L. et al., where intensity of Grem1 expression correlating with the declining left cardiac ventricular function was detected by the immunohistochemistry in the endomyocardial biopsies of patients diagnosed with dilatative cardiomyopathy [24]. Researchers also noticed that 74.8% of selected study cases expressed Grem1 in cardiomyocytes of progressing HF, ranging from very low to very strong intensity of immunohistochemical Grem1 reaction, further emphasizing a significant Grem1 role in the remodeling of cardiomyocytes advancing towards symptomatic HF. Furthermore, Müller I. I. et al. applied Western blot method to confirm that protein levels of Grem1 were the most abundant after 24 h passed after experimental myocardial infarction in mice [26], indicating that effects of Grem1 already manifest at the earliest stages of acute ischemic cardiac muscle injury.

Grem1, a 28 kDa glycosylated protein [40] of Dan protein family of secreted bone matricellular protein (BMP) antagonists, is found in the cellular endoplasmic reticulum and interacts with the elements of the extracellular microenvironment via binding to heparan sulphate proteoglycans in a cell-associated form or resides bound to extracellular glycocalyx close to the protein secretion site near the cellular surface in a secreted form [41,42,43,44,45]. Kaur G. et al. determined that overexpression of Grem1 serves a cardioprotective function by decreasing reactive oxygen species and mitochondrial membrane potential, increasing signaling of NRF/ERK1 pathway as well as the anti-apoptotic proteins expression [46], whereas other researchers indicated the protective importance of angiogenesis while preserving the regular cellular function after exposure to hypoxic environment [26,47,48]. The protective angiogenetic effect of Grem1 is modulated via its dimeric form interacting with VEGFR2 as the agonist of this receptor, as described by Rowan G. C. et al. [47].

Further experimental analysis of myocardial tissue affected by hypoxic conditions revealed that Grem1 is also able to downregulate the profibrotic effects of TGFβ by reducing its functions to activate the collagen synthesis in the myocardial fibroblasts [26], suggesting a more likely complex Grem1 role to maintain the local microenvironmental homeostasis and facilitate the remodeling. Consistent with the protective role of Grem1, Koli K. et al.’s study was able to determine that Grem1 overexpression may lead to a local decrease in the anti-fibrotic Th1 chemokine (CXC10), thus affecting anti-fibrotic chemokine production in pulmonary tissue [18]. Yet, this mechanism of protective Grem1 effect is not characterized in the myocardial tissue.

Moreover, significantly positive correlations between Grem1 expression in cardiomyocytes and the geometric parameters of cardiac myocytes, while the heart muscle is deteriorating towards ischemic HF, were detected in our study. To the best of our knowledge, this is the first time such associative tendencies describing the cardiac myocyte’s geometry changes and Grem1 expression in cardiomyocytes at different stages of ischemic HF are characterized. Documented positive correlations between the changing cardiomyocyte geometry parameters and the overexpression of Grem1 in cardiac myocytes can be explained by Grem1 effect to reactivate cellular embryonic programs in many diseases [18]. The reactivation of the cellular embryonic programs promotes microstructural changes of cardiomyocytes by reorganizing their contractile elements and initiating cytoskeletal rearrangements, serving as a one of the key mechanisms in the cardiac myocyte remodeling [3].

Significantly positive correlation of OPN and Grem1 proteins expression in cardiomyocytes exposed to continuous ischemia documented for the first time by our study suggests possible collaborative role of these proteins within cardiomyocyte during the cellular remodeling when advancing towards ischemic heart disease-induced HF. Still, interaction mechanisms between OPN and Grem1 are not entirely clear. The OPN role in activation of cardiomyocyte hypertrophy-inducing genes expression is documented [33,34], and single cell type RNA expression studies demonstrate that Grem1 is found in the same cluster as the cardiac muscle contractility-coordinating components (troponin T2 of cardiac type, myosin-binding protein C3, and myosin light chain 7) [49]. Therefore, taking our findings on positively correlating expression of OPN and Grem1 into consideration, it can by hypothesized that these proteins may collaborate in regulating the expression of contractile proteins in cardiomyocytes during the cellular remodeling, eventually contributing to ischemic HF. Interestingly, knowing that OPN also synergizes with signaling pathways through epithelial growth factor receptor [50], and Grem1 is an important molecule in adult stem cell control as well as tissue differentiation [51], it can be further hypothesized that both OPN and Grem1 proteins may be involved in regulating cellular adaptive plasticity via changing cellular differentiation mechanisms when myocardial tissue is exposed to continuous ischemia.

Despite the recognized role of OPN and Grem1 proteins expression changes in failing cardiac myocytes contributing to ischemic HF pathogenesis, practical application of OPN and Grem1 protein expression observation in cardiomyocytes during remodeling remains problematic. Lack of standardized uniform criteria and corresponding protein expression data based mostly on experimental animal models create obstacles to assessing the OPN and Grem1 expression in myocardial tissue obtained from the human organism properly [52]. This validated semi-quantitative immunohistochemical study of OPN and Grem1 expression in cardiomyocytes advancing towards ischemic HF in representative human myocardial tissue samples provides an easy reproducible method of immunohistochemical myocardial tissue assessment in clinical setting. Moreover, these data characterizing OPN and Grem1 protein expression in cardiomyocytes of control group set a reference criteria, describing significantly increased OPN and Grem1 protein expression already in stage A ischemic HF, when there are no clinical symptoms of ischemic HF. Therefore, monitoring OPN and Grem1 expression in cardiomyocytes of cardiac muscle tissue fragments (endomyocardial biopsies) can serve as an ancillary diagnostic tool for a clinical follow-up of patients with failing cardiac function before the first symptoms of ischemic HF appear.

The limitations of this study must be stated. OPN and Grem1 expression at the protein level in cardiomyocytes of left cardiac ventricular tissue was analyzed immunohistochemically in our study. We were reluctant to perform mRNA analysis on the archived formalin-fixed paraffin-embedded myocardial tissue. Still, knowing how mRNA is susceptible to degradation, events of RNA fragmentation and RNA cross-linking with tissue proteins during the tissue fixation greatly reduce the amount and quality of extracted RNA, thus misrepresenting OPN and Grem1 mRNA expression in formalin-fixed myocardial tissues.

## 4. Materials and Methods

### 4.1. Study Design and Groups

Myocardial tissue samples from the middle segments of human left cardiac ventricles were selected from the paraffin blocks archive of the Laboratory of Cardiac Pathology of Institute of Cardiology (Lithuanian University of Health Sciences, Kaunas, Lithuania). In total, 82 selected samples of myocardial tissue were further classified into the stage A HF (at-risk for HF), stage B HF (pre-HF), and stages C/D HF (symptomatic and advanced HF, correspondingly) based on the ACC/AHA classification [29].

The demographic and clinical characteristics of the study groups are presented in Table 1. Stage A HF (or at-risk for HF) group was composed of previously healthy male individuals or whose health state had improved or stabilized prior to their death, and who died suddenly due to ischemic heart disease within six hours of experiencing the symptoms of ischemic heart disease [53,54]. No previous HF symptoms were reported for these individuals. A complete postmortem morphological investigation of the heart was performed for them. Acute ischemic injuries of no more than 6 h were documented, and no scars after myocardial infarction were detected in all the cases of this group [55]. The stage B (pre-HF) group was defined by the same characteristics as aforementioned group, except the morphological changes of a scar after myocardial infarction were detected during postmortem morphological investigation of the left cardiac ventricle. The stages C/D HF (symptomatic and advanced HF) group was comprised of male patients who were clinically diagnosed with symptomatic or advanced ischemic HF classified as stage C or D according to ACC/AHA classification [29], a surgical procedure of a heart transplantation was performed for them, and a complete morphological investigation of the explanted heart was carried out.

Male patients who died from the external causes or acute non-cardiovascular diseases, and their hearts were examined during postmortem morphological investigation were selected as a control group.

No other diseases or conditions, such as systemic arterial hypertension, congenital or acquired cardiac valve disease, cardiomyopathy, diabetes mellitus or pulmonary diseases, that could lead to the heart’s remodeling were diagnosed for all these patients. A comprehensive histological examination was performed for all selected cases before an immunohistochemical study. Therefore, samples of myocardial tissue from the middle segments of the free wall of left cardiac ventricles in all selected cases were extracted with special attention to avoiding the areas affected by acute ischemic injuries or scarring after myocardial infarction.

### 4.2. Immunohistochemistry

An immunohistochemical approach with independent antibody validation strategy in accordance with recommendations by the International Working Group for Antibody Validation was adopted to evaluate expression of OPN and Grem1 in cardiomyocytes in ischemic HF [57,58,59].

Selected formalin-fixed paraffin embedded myocardial tissue samples were cut into 3 µm microsections applying a Leica RM2235 microtome (Leica Biosystems, Deer Park, IL, USA), mounting these microsections on Menzel SuperFrost Plus slides (Menzel, Braunschweig, Germany) afterwards. The slides were left to air-dry at room temperature overnight and baked at 50 °C for a minimum of 12 h. Deparaffinization by xylene, immersion in decreasing concentrations of ethyl alcohol and rehydration with distilled water procedures were performed.

Antigen retrieval was performed applying a microwave tissue processor RHS-1 (Milestone Medical, Roseland, NJ, USA) and incubating the samples in TRIS/EDTA buffer (Target Retrieval Solution, pH 9.0, Agilent Technologies Inc., Wood Dale, IL, USA) at 110 °C for 8 min. Immunohistochemical staining was performed using “Shandon Coverplate” plates (Thermo Fisher Scientific, Waltham, MA, USA). Endogenous peroxidase was blocked.

Two distinct primary antibodies were applied to assess OPN expression in cardiomyocytes: monoclonal mouse anti-human OPN (MPIIIB10(1)) antibody (Developmental Studies Hybridoma Bank, Iowa City, IA, USA) and polyclonal rabbit anti-human OPN (PA5-13494) antibody (Thermo Fisher Scientific, Waltham, MA, USA) (Table 2). The aforementioned strategy was also applied to evaluate Grem1 expression in cardiomyocytes: polyclonal rabbit anti-human Grem1 (ab22138) antibody (Abcam, Cambridge, UK) and polyclonal rabbit anti-human Grem1 (GTX03394) antibody (GeneTex, Irvine, CA, USA).

Primary antibodies of OPN and Grem1 were diluted with Antibody Diluent (Agilent Dako, S080983, Santa Clara, CA, USA) by applying appropriate proportions of dilution (see Table 2). They were incubated at room temperature for 60 min. Afterwards, EnVision FLEX+ visualization system with Mouse or Rabbit Linker and HRP Magenta chromogen (Agilent Dako, K800221-2, K802121-2, GV92511-2, Santa Clara, CA, USA), following the manufacturer’s instructions, was applied. Procedures of hematoxylin counterstaining (Agilent Dako, S330930-2, Santa Clara, CA, USA), dehydration by increasing concentrations of ethyl alcohol and xylene were performed before permanent coverslip using a polystyrene coating material.

The tissue fragments of breast carcinoma served as a positive control for anti-human OPN antibody, whereas kidney tissue samples served as a positive control for anti-human Greml antibody. The same protocols of immunohistochemical reactions were applied in all these control tissues as in analyzed myocardial samples, running these immunohistochemical reactions simultaneously. This step was performed to ensure the specificity of the primary antibodies. The IgG of the same isotype as the primary antibody dilution served as a reagent control, not yielding any specific staining.

### 4.3. Analysis of OPN and Grem1 Immunostaining

A semi-quantitative evaluation for OPN and Grem1 expression in cardiomyocytes was applied by using a score evaluation for the immunohistochemical reaction intensity: 0 points = not detected, 1 point = weak, 2 points = moderate, 3 points = strong. The results of each immunohistochemical reactions were observed in the 50 microscopic fields at 40× magnification (light microscopy, motorized microscope Olympus BX51, Olympus Corporation, Tokyo, Japan) for each selected case, indicating the amount of cardiomyocytes within each category of grading scale separately in the selected case according to the study design (% of all the longitudinal plane cardiomyocytes per case). A formula to evaluate the overall scope of the intensity of the immunohistochemical reactions in the selected myocardial tissue was applied: immunostaining score = (1 point × amount of the representative cardiomyocytes %) + (2 points × amount of the representative cardiomyocytes %) + (3 points × amount of the representative cardiomyocytes %)/10.

Also, analysis of the immunohistochemical reactions against OPN and Grem1 was performed applying a stratification system, based on the workflow outlined in the Human Protein Atlas [60]: 0 = not detected (negative or weak staining in less than 25% of cardiomyocytes); 1 = low (weak staining in at least 25% of cardiomyocytes and moderate staining in less than 25% of cardiomyocytes); 2 = medium (moderate staining in at least 25% of cardiomyocytes or strong staining in less than 25% of cardiomyocytes), 3 = high (strong staining of at least 25% cardiomyocytes). The categories characterized on the workflow by the Human Protein Atlas were identified for each selected case in the stage A HF, stage B HF, stages C/D HF, and control groups.

Results of OPN and Grem1 immunohistochemical reactions applying the independent antibody validation strategy in accordance with recommendations by the International Working Group for Antibody Validation [57,58,59] revealed no significant differences when analyzing the immunohistochemical reactions with the alternative immunogens of OPN and Grem1 antibodies, concluding that the immunohistochemical study had the enhanced reliability score [60]. The evaluation was performed by two researchers independently, and none of these researchers knew what the groups of selected cases during this process of evaluation were. The inter-observer and intra-observer variability was evaluated by Kappa (κ) statistics (*Cohen*’s κ coefficient > 0.9).

### 4.4. Statistical Analysis

Normality of continuous variables distribution was assessed with the Shapiro-Wilk tests. Continuous variable that fits normality distribution was reported as mean (standard deviation or standard error). Statistically significant differences between the stage A HF, stage B HF, stages C/D HF, and control groups were determined by ANOVA with post hoc Bonferroni tests for multiple comparisons. Pearson’s correlation was applied for evaluating correlation trends. Values of p < 0.05 were considered statistically significant. Statistical analysis was performed by Statistical Package for the Social Sciences (SPSS) software (SPSS Statistics version 29.0, IBM, Armonk, NY, USA).

## 5. Conclusions

A significant increase of OPN and Grem1 proteins expression in cardiomyocytes, already observed in the stage A HF (or at-risk for HF) group, was documented in our study by immunohistochemical analysis, continuing to increase in the stage B HF (or pre-HF) group, and achieving the peak expression in the stages C/D HF. These novel findings, together with the characterized positive correlations between the changes of OPN and Grem1 expression in cardiac myocytes and their cellular geometry suggest that OPN and Grem1 are involved in rearranging cardiomyocyte’s geometry and possibly functions of intracellular contractile elements from the earliest stage of cardiac myocyte remodeling, providing a new insight into the pathogenesis mechanism of ischemic HF.

Considering the role of OPN and Grem1 in the pathogenesis mechanisms of ischemic heart disease-induced HF, targeting OPN and Grem1 as the possible diagnostic and therapeutic agents can create an alternative option in the attempts to optimize the individual patient-focused healthcare strategy for ischemic HF patients. Also, our data provide a validated source for ongoing studies in the *OPN* and *Grem1* genes translation, diagnostic and therapeutic HF targets research, thus extending the overall fundamental knowledge on the remodeling processes in cardiomyocytes and HF pathogenesis.

## Figures and Tables

**Table 1:** Characteristics of the study population by groups.

Characteristic	Control Group n = 23	Stage A HF Group n = 25	Stage B HF Group n = 23	Stages C/D HF Group n = 34
Age (SD), years	50.5 (7.9)	53.8 (8.0)	54.4 (7.7)	56.2 (7.2)
Sex	Male	Male	Male	Male
Previous clinical symptoms of HF	No	No	No	Yes
Atherosclerotic stenosis ≥ 75% in at least one coronary artery	No	Yes	Yes	Yes
Scar after myocardial infarction	No	No	Yes	Yes
Mean length (SD) of left ventricular cardiomyocyte, µm [56]	61.8 (6.3)	72.2 (5.4)	78.9 (6.3)	103.3 (10.7)
Mean diameter (SD) of left ventricular cardiomyocyte, µm [56]	11.7 (1.5)	14.3 (1.0)	15.2 (1.3)	18.9 (2.7)

Abbreviations: HF—heart failure; stages A, B, C, D of HF—according to ACC/AHA classification; SD—standard deviation.

**Table 2:** Characteristics of the primary antibodies used for immunohistochemistry.

Antibody	Species	Immunogen	Dilution	Manufacturer (Catalog Number)	RRID	Lot Number
Osteopontin (OPN)	Mouse monoclonal	Bone protein fractions, immunogen sequence—full length	1:100	Developmental Studies Hybridoma Bank, DSHB (MPIIIB10(1))	AB_2286610	-
Osteopontin (OPN)	Rabbit polyclonal	KLH conjugated synthetic peptide corresponding to amino acids 273 through 301 from the C-terminal region of human SPP1	1:50	Thermo Fisher Scientific (PA5-13494)	AB_2286594	YI4041893
Gremlin 1 (Grem1)	Rabbit polyclonal	Synthetic peptide, corresponding to amino acids from 52 through 67	1:100	Abcam (ab22138)	AB_446814	GR3362005-1
Gremlin 1 (Grem1)	Rabbit polyclonal	KLH conjugated synthetic peptide derived from human gremlin 1, corresponding to amino acids from 101 through 184	1:200	GeneTex (GTX03394)	-	822303719

Abbreviations: OPN—osteopontin; Grem1—gremlin 1; KLH—keyhole limpet hemocyanin; SPP1—secreted phosphoprotein 1; DSHB—Developmental Studies Hybridoma Bank; RRID—Research Resource Identifier.

## Data Availability

The data presented in this study are available from the corresponding author upon reasonable request.

## Abbreviations

ACC/AHA: American College of Cardiology/American Heart Association; ANOVA: analysis of variance; β2AR: beta 2 adrenergic receptor; BMP: bone matricellular protein; cAMP: cyclic adenosine monophosphate; CD44: cluster of differentiation 44; CXC10: C-X-C motif chemokine 10; DSHB: Developmental Studies Hybridoma Bank; Epac1: exchange factor activated by cAMP1; ERK1-2: extracellular signal-regulated kinase 1-2; Grem1: gremlin 1; HF: heart failure; HRP: horseradish peroxidase; Ig: immunoglobulin; kDa: kilodalton; KLH: keyhole limpet hemocyanin; MEK1-2: mitogen-activated protein kinase 1-2; NFAT: nuclear factor of activated T-cells; NRF: nuclear respiratory factor; NYHA: New York Heart Association; OPN: osteopontin; RAF kinase: rapidly accelerated fibrosarcoma kinase; RAS protein: rat sarcoma virus protein; RRID: research resource identifier; SD: standard deviation; SPP1: secreted phosphoprotein 1; TGFβ: transforming growth factor beta; VEGFR2: vascular endothelial growth factor receptor 2.
